# Honey Bee Viromes From *Varroa destructor* ‐Resistant and Susceptible Colonies

**DOI:** 10.1111/1758-2229.70097

**Published:** 2025-05-10

**Authors:** Daniela Arredondo, Sofia Grecco, Yanina Panzera, Pablo Zunino, Karina Antúnez

**Affiliations:** ^1^ Laboratorio de Microbiología y Salud de las Abejas, Departamento de Microbiología, Instituto de Investigaciones Biológicas Clemente Estable Ministerio de Educación y Cultura Montevideo Uruguay; ^2^ Centro de Investigación en Ciencias Ambientales (CICA), Instituto de Investigaciones Biológicas Clemente Estable Ministerio de Educación y Cultura Montevideo Uruguay; ^3^ Programa de Desarrollo de las Ciencias Básicas (PEDECIBA) Ministerio de Educación y Cultura ‐ Universidad de la República Montevideo Uruguay; ^4^ Sección Genética Evolutiva, Departamento de Biología Animal, Facultad de Ciencias Universidad de la República Montevideo Uruguay; ^5^ Plataforma Genómica, Facultad de Ciencias Universidad de la República Montevideo Uruguay

**Keywords:** honey bee viral communities, honey bee virome, *Varroa destructor* ‐resistant colonies, *Varroa destructor* ‐susceptible colonies

## Abstract

Honey bees ( *Apis mellifera* ) play a crucial role in global food production through pollination services. However, their populations are threatened by various stressors, like the ectoparasitic mite *Varroa destructor* and associated viral pathogens. In this study, we aimed to characterise and compare the viral communities (viromes) in *V. destructor* ‐resistant and susceptible colonies using high‐throughput sequencing. Our findings revealed differences in virome composition associated with the season and not with the resistance or susceptibility to *V. destructor* . Furthermore, we detected *Apis mellifera* filamentous virus (AmFV) and Lake Sinai virus (LSV) for the first time in Uruguay, and obtained the complete or partial genomes of both viruses, along with those of other previously described viruses, such as Acute bee paralysis virus (ABPV), Black queen cell virus (BQCV), Deformed wing virus (DWV), and Sacbrood virus (SBV). This study contributes to a deeper understanding of the virome dynamics in honey bees. It highlights the importance of this type of study for the early detection of new viral pathogens, which could help to understand the tripartite network involving *V. destructor* , honey bees, and viruses.

## Graphical abstract

Our study reveals seasonal variations in honey bee virome composition among colonies independent of their *Varroa destructor* susceptibility. We report the first detection of AmFV and LSV in Uruguay, obtaining complete or partial genomes of multiple viruses. These findings enhance virome understanding and highlight the importance of early pathogen detection.

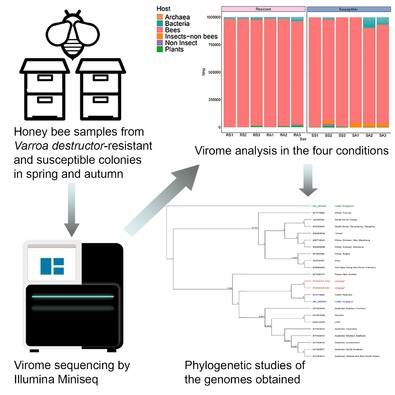

## 1 Introduction

Pollinators are essential for the reproduction of both crops and wild plant species (Potts et al. 2016). Among them, the honey bee ( *Apis mellifera* ) is the most economically significant, contributing to global food production by visiting more than 90% of the 107 most important crop types (Klein et al. 2007).

Honey bees are native to Africa, Europe, and the Middle East. They have been introduced to Oceania, Asia, and America in several waves, mainly due to honey production and their importance in pollination (Visick and Ratnieks 2023). In recent decades, large‐scale honey bee colony losses have been reported worldwide (Neumann and Carreck 2010; Gray et al. 2023; Aurell et al. 2024; Requier et al. 2024). These losses are linked to biotic and abiotic stress factors. Among the biotic stress factors, the mite *Varroa destructor* and viral pathogens are among the most significant threats to honey bees' health (Goulson et al. 2015; Steinhauer et al. 2018; Traynor et al. 2020). The mite feeds on the fat bodies of honey bee pupae, reducing their lifespan, immunosuppressing honey bees, and facilitating infections by pathogens (Yang and Cox‐Foster 2005; Rosenkranz et al. 2010; Ramsey et al. 2019). This parasite is a highly efficient vector of honey bee viruses, driving changes in virus distribution, prevalence, and virulence (Rosenkranz et al. 2010; Beaurepaire et al. 2020; Traynor et al. 2020). Over 80 viruses have been described to infect honey bees, primarily positive‐strand RNA viruses from the *Dicistroviridae* and *Iflaviridae* families. Some studies suggest that certain viruses, for example, Black queen cell virus (BQCV) or Deformed wing virus variant B (DWV‐B), can reproduce on the mite, even though there is no general agreement on this aspect (Ongus et al. 2004; Annoscia et al. 2019; Sabahi et al. 2020; Damayo et al. 2023). Typically, honey bee colonies harbour multiple viruses, and their infection levels can fluctuate throughout the year (Beaurepaire et al. 2020). Viral infections can affect honey bees in several ways, causing spasms, tremors, impaired cognitive abilities, and defective wing development. These infections can also result in the death of developing larvae or pupae and lead to a reduced lifespan in adult honey bees (Beaurepaire et al. 2020).

Beekeepers usually use a range of chemical treatments (based on synthetic and organic acaricides) to treat colonies infested with *V. destructor* (Rosenkranz et al. 2010; Jack and Ellis 2021). However, more sustainable approaches to pest management, which could include the administration of beneficial microbes (Tejerina et al. 2020; Vilarem et al. 2021; Arredondo et al. 2023) or mechanical control techniques, are being studied (Jack and Ellis 2021).

Some honey bee populations can survive without acaricide treatments (Locke 2016; Mendoza et al. 2020; Moro et al. 2021). These *V. destructor* ‐resistant populations could exhibit a high hygienic behaviour, grooming, smaller colony size, reduced breeding time, or the suppression of mite reproduction (Locke 2016). Moreover, the genetic selection for *V. destructor‐*surviving populations may also confer resistance to honey bee viruses, as evidenced by colonies with lower DWV levels and higher levels of immune‐related genes (Locke 2016).

Recently, Mendoza et al. (2020) confirmed the existence of a *V. destructor‐*resistant population in northeastern Uruguay, South America. This population showed higher hygienic and grooming behaviours and a lower level of DWV infection compared to mite‐susceptible colonies (Mendoza et al. 2020). Later, Invernizzi et al. (personal communication) continued monitoring those colonies, analysing varroa and viral dynamics through qPCR. However, there is scarce knowledge about the relationship with other viruses in those colonies. In this study, we aimed to characterise and compare the viral communities (viromes) in *V. destructor* ‐resistant and susceptible colonies. Studying the interaction between *V. destructor* , honey bees, and their viromes is crucial for enhancing colony resilience and safeguarding the health and survival of honey bee populations.

## 2 Experimental Procedures

### 2.1 Bee Samples

Bee samples were collected in the framework of a previous study (ECOS‐SUD program, led by Dr. Ciro Invernizzi and Dra. Anne Dalmon) and were stored at −80°C. Bees belong to *V. destructor* ‐susceptible (S) and resistant (R) colonies located in Treinta y Tres department (−33°15′20.147″S, 54°25′37.081″W), northeastern Uruguay. Susceptible colonies were treated with flumethrin the previous autumn. In December (late spring), all colonies harboured *V. destructor* , with infestation levels of 5.25% in resistant colonies and 3.56% in susceptible colonies. By March, infestation remained similar in resistant colonies (4.95%) but increased to 15.1% in susceptible colonies. No virus‐related symptoms, such as deformed wings, were observed. Nurse honey bees were sampled in December (late spring) and March (early autumn) from six *V. destructor‐resistant* (R) and six susceptible (S) colonies. Honey bees were sacrificed and stored at −80°C until analysis.

Samples were pooled in groups of 2 colonies (20 individuals per colony) per colony type (R/S) and per season (S/A), reaching a total of 12 pools: 3 of resistant colonies in spring (RS), 3 of resistant colonies in autumn (RA), 3 of susceptible colonies in spring (SS), and 3 of susceptible colonies in autumn (SA).

### 2.2 RNA Extraction, Enrichment, and Sequencing

Enrichment of viral particles and sequencing were carried out using a method developed in the Plataforma de Genómica de Facultad de Ciencias, Universidad de la República, Uruguay (Fuques Villalba 2021). Honey bees were homogenised in 5 mL of 1X PBS and centrifuged at 1550 × g for 15 min at 4°C. The supernatant was collected and filtered through a 0.40 nm filter to remove cell‐sized particles and bacteria. The filtrates were ultracentrifuged at 99801.5 × g for 2:30 h at 4°C in a 30% sucrose cushion. The pellet was resuspended in RNAase‐free water and treated with a nuclease mix. Then, RNA was purified using PureLink Viral RNA/DNA Mini Kit (InvitrogenTM) and used to generate double‐stranded cDNA (ds‐cDNA) using a ds‐cDNA synthesis kit (Maxima H Minus, Thermo Fisher Scientific) with random primers. The obtained ds‐cDNA was amplified using a REPLI‐g Minikit (Qiagen, Germany), followed by RNA purification and quantification using beads (VAHTS RNA Clean Beads, Vazyme, China) and a Qubit fluorometer (Thermo Fisher Scientific, USA). Subsequently, 100 ng of ds‐cDNA was used to prepare the library, with Nextera DNA flexible library preparation kit (Illumina, USA) with dual indexing. Quality control was performed on a Fragment Analyser 5200 system (Agilent Technologies, USA) using a standard NGS sequencing analysis kit (Agilent Technologies, USA). Libraries were sequenced using Illumina MiniSeq (300 cycles, paired‐end × 150 bp) following standard Illumina protocols.

### 2.3 Reads Filtering and De Novo Assembly

The raw reads were demultiplexed automatically on the MiniSeq platform with the default settings. In Ubuntu v.20.04.6, adapter and quality trimming were performed with Trim Galore v0.6.4, using default parameters. Subsequently, using the Burrows‐Wheeler Aligner pipeline (BWA; Li 2013), the reads were mapped against the host reference genome (*A. mellifera*, Genome assembly: GCA_003314205), and those that did not map to the reference were filtered and selected. The resulting reads were assembled *de novo* using SPAdes v 3.12.0 (Bankevich et al. 2012). The resulting contigs were classified using the DIAMOND BLASTx (v.2.0.5) algorithm against the non‐redundant (nr) protein from RefSeq (Buchfink et al. 2015). Finally, DIAMOND results were filtered using awk, keeping only the hits with a bitscore higher than 200 and a percentage of identity higher than 50% and extracted for statistical analysis.

### 2.4 Statistical Analysis of Viral Operational Taxonomic Units (vOTUs)

The DIAMOND results and metadata were imported, analysed, and visualised in R studio v2023.03.0.386 (R Core Team 2023) using many packages, including tidyverse v2.0.0 (Wickham et al. 2019) and phyloseq v1.44.0 (McMurdie and Holmes 2013). The viral transcripts per million (vTPMs) were calculated, and those with at least 1% relative abundance in a minimum of 1 sample were retained using the ‘filterfun_sample’ function on the Genefilter package v1.74.0 (Gentleman et al. 2021). Next, alpha and beta diversity were calculated using the Vegan package v2.5–7 (Oksanen et al. 2013). The alpha diversity was calculated with the number of observed viral TPMs and the Shannon index using the ‘estimate_richness’ function (Oksanen et al. 2013). Then, beta diversity was evaluated by Bray‐Curtis, Jaccard, UniFrac weighted (by the relative abundance of ASVs), and UniFrac unweighted indexes (Oksanen et al. 2013). Permutational multivariate analysis of variance with the ‘adonis’ function was used to test the effect of treatments on community structure on beta diversity data. We then used the function ‘betadisper’ to test for homogeneity of multivariate dispersions (Anderson 2006; Anderson et al. 2006) and compared the distances of individual samples to group centroids in multidimensional space using ‘permutest’.

### 2.5 Genome Assembly and Phylogenetic Trees

The viral reads were imported, trimmed for each library (QC ≥ 30; minimum length of 60 bp), and mapped to various viruses to obtain complete or partial genomes using the Minimap2 mapper (Short reads) available in the Geneious Prime 2024.0.5 software (Kearse et al. 2012). We included Acute bee paralysis virus (ABPV), Aphid lethal paralysis virus (ALPV), Black queen cell virus (BQCV), Israeli acute paralysis virus (IAPV), and Kashmir bee virus (KBV) from the *Dicistroviridae* family, Deformed wing virus (DWV), Sacbrood virus (SBV), and *Varroa destructor* virus‐2 (VDV‐2) from the *Iflaviridae* family; Bee macula‐like virus (BeeMLV) from the *Tymoviridae* family; Lake Sinai Virus (LSV) from the Sinhaliviridae family, *Apis mellifera* filamentous virus (AmFV) from the *Baculoviridae* family and finally Chronic bee paralysis virus (CBPV), which is still unclassified.

Sequence alignments were carried out on those whose complete or partial genomes were obtained, and maximum‐likelihood phylogenetic trees were built with a 1000‐replicate bootstrap using MAFFT and FastTree plugins with default settings in Geneious Prime 2024.0.5 software.

## 3 Results

### 3.1 Virome Composition of *V. destructor* ‐Resistant and Susceptible Colonies

**FIGURE 1:**
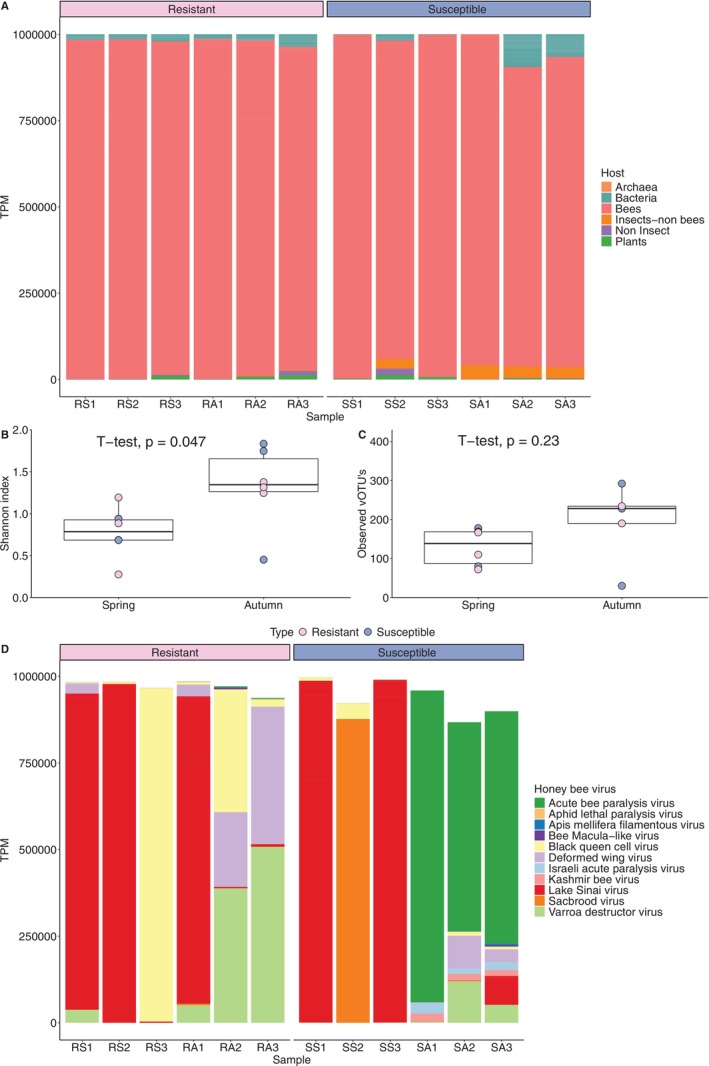
Virome composition in *Varroa destructor‐*resistant and susceptible honey bee colonies. Six colonies per colony type were sampled in spring and autumn and pooled into groups of two. (A) Transcripts per million reads (TPM) of viruses associated with different hosts per colony (B) Shannon index, (C) Observed vOTU's, (D) Transcripts per million reads of viruses associated with honey bees in each colony.

From the 12 pooled samples analysed, an average of 7,282,131 reads/library was obtained, and the average number of reads that passed the QC filter per library was 7,213,408 (Table S1). Viruses described for archaea, bacteria, honey bees, insects‐non bees, non‐insects, and plants were identified (Figure 1A).

*Varroa destructor* ‐resistant and susceptible honey bees harbour similar viral richness and diversity through each season (Shannon and Observed vOTU's index: *t* test *p* > 0.05 in both cases).

On the other hand, the season influenced the diversity of those viromes. Autumn honey bees harboured a higher viral diversity than in spring (Shannon index ranged from 0.45 to 1.83 in autumn vs. 0.2 to 1.2 in spring, *t*‐test, *p* = 0.047, Figure 1B), although richness was similar in both groups (30 to 433 in autumn vs. 72 to 178 in spring, *t* test, *p* = 0.23), (Figure 1C).

PERMANOVA analysis confirmed that the season affects the composition of honey bee viromes (Comparison between the four groups: Adonis‐Bray test; *p* = 0.02, *R* ^2^ = 0.33); Pairwise comparison spring vs. autumn colonies (Adonis‐Bray test; *p* = 0.01, *R* ^2^ = 0.14); Pairwise comparison resistant vs. susceptible colonies (Adonis‐Bray test; *p* = 0.37, *R* ^2^ = 0.09).

### 3.2 Characterisation of Viruses Associated With Honey Bee Diseases

Transcripts from 10 viral species of positive‐sense single‐stranded (+SS) RNA viruses and one double‐stranded (ds) DNA virus were identified in the analysed colonies: five belong to the *Dicistroviridae* family ABPV, ALPV, BQCV, IAPV, and KBV; two belong to the *Iflaviridae* family, DWV and SBV and one belongs to the *Tymoviridae* family (BeeMLV, Figure 1D).

When analysing only the viruses known to be linked with honey bee diseases, *V. destructor* ‐resistant and susceptible honey bees also harbour similar viral richness and diversity. The season did not influence those parameters (Shannon and Observed vOTU's index: *t* test, *p* > 0.05 in all cases). The only honey bee‐specific virus with significant differences between groups was ABPV (Figure S1). The transcripts per million obtained from the susceptible colonies in autumn were significantly higher than in spring (*t* test, *p* = 0.03, Figure S1A). A significant number of transcripts per million were also observed in susceptible colonies in autumn compared to resistant colonies (*t* test, *p* = 0.015, Figure S1B).

### 3.3 Whole‐Genome Analysis and Phylogenetic Relationships

Several complete or partial genomes of different viruses were obtained: 5 for ABPV, 2 for AmFV, 12 for BQCV, 7 for DWV, 7 for LSV, and 2 for SBV. It was not possible to obtain the complete genome of ALPV, BeeMLV, IAPV, KBV, or VDV‐2. The transcripts mapping IAPV and KBV are also mapped with ABPV, and the ones that mapped VDV‐2 also mapped with DWV, suggesting they were wrongly automatically classified.

**FIGURE 2:**
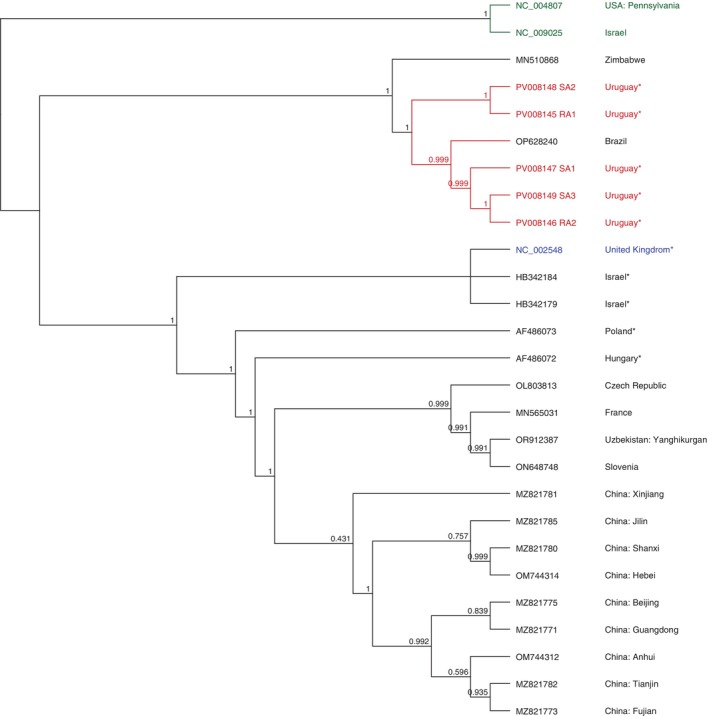
Phylogenetic dendrogram based on the Acute bee paralysis virus (ABPV) complete genomes using FastTree 2.1: Approximately‐Maximum‐Likelihood Trees for Large Alignments algorithm. Uruguayan genomes (red), IAPV (NC_009025) and KBV (NC_004807) as outlier group (green), reference genome (blue), and the rest of the sequences (black). All sequences used for comparison were obtained from the NCBI database (accession numbers are indicated). For cases where the country information was not automatically associated with the sequence, the corresponding publication describing it was consulted. These countries are marked with an asterisk.

ABPV complete and partial genomes were obtained from resistant and susceptible colonies in autumn (SA1, SA2, SA3, RA1, RA2). The genomes showed a percentage of identity ranging from 82.9% to 83.4% with the ABPV reference genome (GenBank accession NC_002548). However, they showed a higher percentage of identity with a sequence from Brazil (96.2% to 97.3%, coverage 82.1%–99.9%, GenBank accession OP628240.1). Phylogenetic reconstruction and bootstrapping analysis clustered Uruguayan genomes with sequences from Brazil and Zimbabwe (GenBank accession MN510868), and separately from the rest of ABPV complete genomes available (from the Czech Republic, China, France, Hungary, Poland, Slovenia, the United Kingdom, Uzbekistan and the reference genome, Figure 2).

**FIGURE 3:**
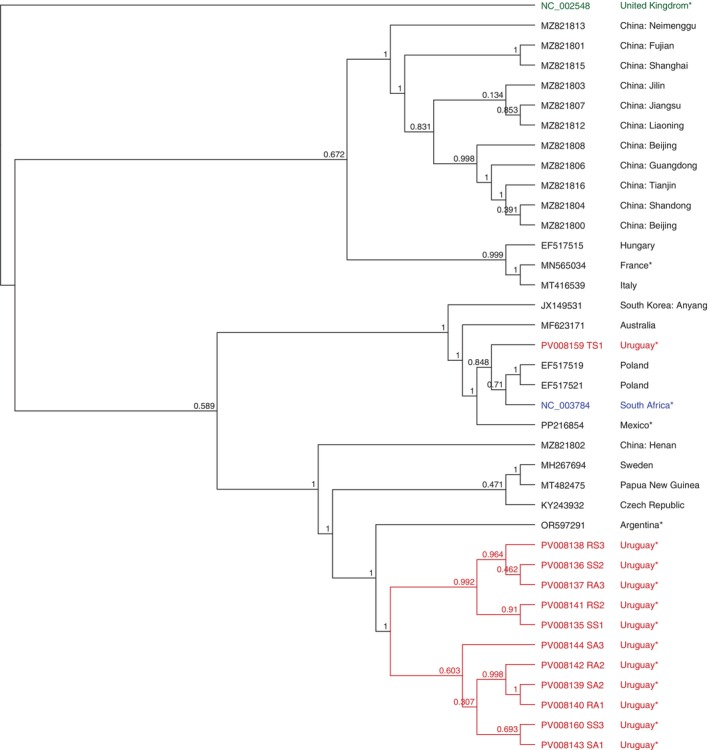
Phylogenetic dendrogram based on the Black queen cell virus (BQCV) complete and partial genomes using FastTree 2.1: Approximately‐Maximum‐Likelihood Trees for Large Alignments algorithm. Uruguayan genomes (red), ABPV (NC_002548) as an outlier group (green), reference genome (blue), and the rest of the sequences (black). All sequences used for comparison were obtained from the NCBI database (accession numbers are indicated). For cases where the country information was not automatically associated with the sequence, the corresponding publication describing it was consulted. These countries are marked with an asterisk.

BQCV complete and partial genomes were obtained from spring and autumn pools in resistant and susceptible colonies (SS1, SS2, SS3, RS1, RS2, RS3, SA1, SA2, SA3, RA1, RA2, RA3). The genomes showed 83.82% to 87.81% identity with the reference genome (GenBank accession NC_003784). However, they showed higher identity with a sequence from Sweden (80.13% to 95.63% of identity, covering 71.6%–100% of the genome, GenBank accession MH267694). Phylogenetic reconstruction and bootstrapping analysis divided the Uruguayan strains into two clusters, one including the sequence from the RS1 group that is similar to the reference genome and sequences from Poland and the reference genome (GenBank accession EF517519, EF517521, NC_003784, Figure 3). The second clade comprises the other 11 Uruguayan sequences and a sequence from Argentina (GenBank accession OR597291). This group clustered with sequences from the Czech Republic, Papua New Guinea and Sweden (GenBank accession KY243932, MT482475 and MH267694, respectively, Figure 3).

**FIGURE 4:**
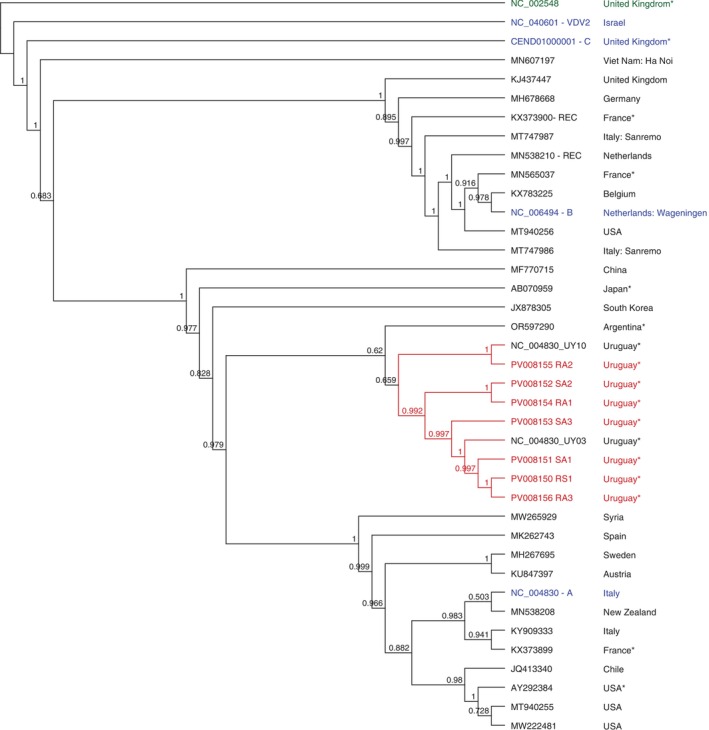
Phylogenetic dendrogram based on the Deformed wing virus (DWV) complete genomes using FastTree 2.1: Approximately‐Maximum‐Likelihood Trees for Large Alignments algorithm. Uruguayan genomes (red), ABPV (NC_002548) as an outlier group (green), reference genome (blue), and the rest of the sequences (black). All sequences used for comparison were obtained from the NCBI database (accession numbers are indicated). For cases where the country information was not automatically associated with the sequence, the corresponding publication describing it was consulted. These countries are marked with an asterisk.

The DWV complete and partial genomes were obtained from the resistant and susceptible colonies in autumn and spring. These genomes showed a high percentage of identity (96.5% to 97.8%) with the reference genome from the DWV‐A from Italy (GenBank accession NC_004830), with a coverage of 100% in all cases. Phylogenetic analysis revealed that the seven Uruguayan sequences grouped closely with a DWV sequence from Argentina (GenBank accession OR597290) and with two DWV sequences obtained from Uruguayan *V. destructor* mites (Hasegawa et al. 2023). In contrast, they exhibited a distant relationship to a sequence originating from Chile (GenBank accession JQ413340, Figure 4), separately from the branches that contain the DWV‐B and DWV‐C sequences (GenBank accession NC_006494 and CEND01000001, respectively, Figure 4).

**FIGURE 5:**
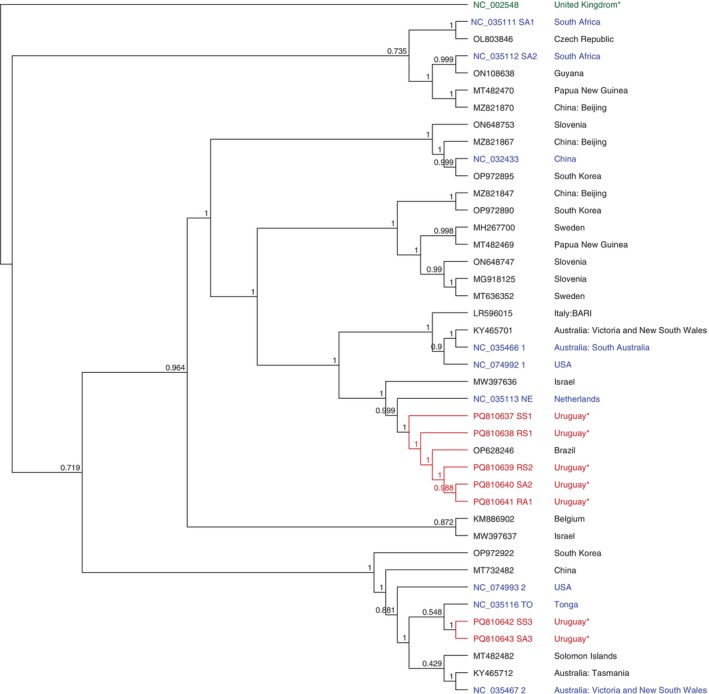
Phylogenetic dendrogram based on the Lake Sinai virus (LSV) complete genomes using FastTree 2.1: Approximately‐Maximum‐Likelihood Trees for Large Alignments algorithm. Uruguayan genomes (red), ABPV (NC_002548) as an outlier group (green), reference genome (blue), and the rest of the sequences (black). All sequences used for comparison were obtained from the NCBI database (accession numbers are indicated). For cases where the country information was not automatically associated with the sequence, the corresponding publication describing it was consulted. These countries are marked with an asterisk.

Complete and partial genomes from LSV sequences were obtained from resistant and susceptible colonies in autumn and spring (SS1, SS3, RS1, RS2, SA2, SA3, RA1). Obtained genomes showed a percentage of identity of 69.96% to 86.36% and a coverage of 95.2% to 99.9% with the reference genome (GenBank accession NC_035113). The genomes from pools SS3 and SA3 revealed a percentage of identity of 90.2% and a coverage of 99.9% with the reference genome (GenBank accession NC_035116). The genomes from pools RS1, RS2, SA2, and RA1 revealed a percentage of identity ranging from 89.01% to 95.06% and a coverage ranging from 96.7% to 100% with a Brazilian genome (GenBank accession OP628246). Phylogenetic reconstruction and bootstrapping analysis grouped the Uruguayan strains into two clusters, one with the sequences from the genomes from pools SS3 and SA3 that cluster with the LSV‐TO variant from Australia, China, Tonga, and USA sequences (GenBank accession NC_035467, MZ821886, NC_035116 and NC_074993 respectively, Figure 5). The second clade comprises the rest of the genomes and includes sequences from the LSV‐NE variant from Brazil, Netherlands, and Israel (GenBank accession OP628246, NC_035113, and MW397636, respectively, Figure 5).

**FIGURE 6:**
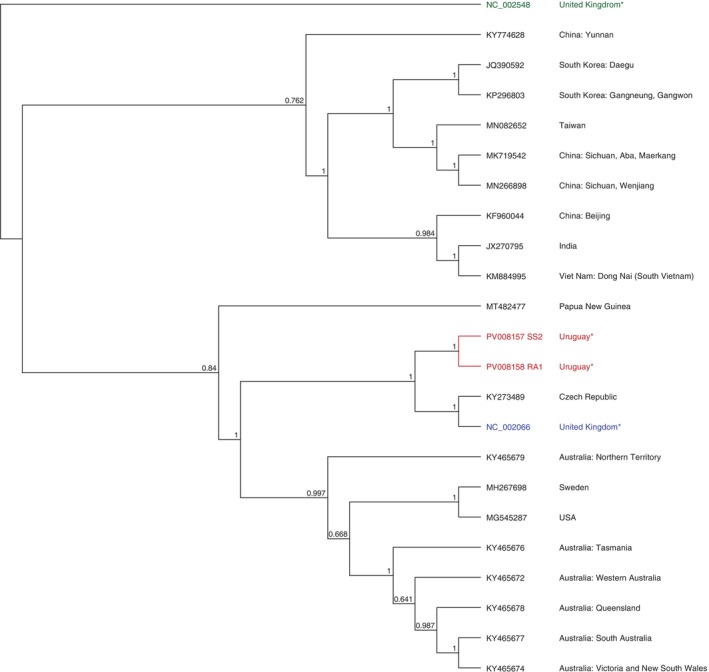
Phylogenetic dendrogram based on the Sacbrood virus (SBV) complete genomes using FastTree 2.1: Approximately‐Maximum‐Likelihood Trees for Large Alignments algorithm. Uruguayan genomes (red), ABPV (NC_002548) as an outlier group (green), reference genome (blue), and rest of the sequences (black). All sequences used for comparison were obtained from the NCBI database (accession numbers are indicated). For cases where the country information was not automatically associated with the sequence, the corresponding publication describing it was consulted. These countries are marked with an asterisk.

Regarding SBV, one partial genome was obtained from a resistant colony in autumn (RA1) and one complete genome from a susceptible pool of colonies in spring (SS2). The genomes showed a high similarity (94.5%) and coverage (98.1% and 100%) with the reference genome (GenBank accession NC_002066). Uruguayan sequences grouped with the reference genome from the United Kingdom and a sequence from the Czech Republic (GenBank accession KY273489), being more closely related to European sequences than to Asian ones (Figure 6).

Finally, two partial sequences were obtained for AmFV in autumn, one from a pool of resistant and one from susceptible colonies (RA1 and SA3). The sequences showed 84.06% (RA1) and 83.31% (SA3) identity with the reference genome of AmFV from Switzerland (GenBank accession NC_027925), and coverage of 35.6% and 32.1%, respectively. As there are just three partial genomes available on the NCBI database, no phylogenetic analyses were performed.

## 4 Discussion

*Varroa destructor* is the main biotic threat that challenges honey bee health. Besides the direct damage to those insects, it is a vector for viruses, which can also negatively impact honey bee development, behaviour and lifespan (Beaurepaire et al. 2020). The hive and its environment drive colonies' virome composition, and infestation by *V. destructor* alters both the viral composition of hives and the virulence of associated viruses (Beaurepaire et al. 2020; Kadlečková et al. 2022; Warner et al. 2023). To mitigate colony losses, beekeepers rely on acaricides to control mite populations. While these acaricides are highly effective, the emergence of resistant *V. destructor* populations has been reported, limiting their long‐term efficacy (Mitton et al. 2022). This highlights the need for alternative strategies to manage mites and support sustainable beekeeping. One alternative to minimise the use of acaricides is focused on identifying *V. destructor‐*resistant honey bee populations (Locke 2016; Mondet et al. 2020; Morfin et al. 2024). In Uruguay, where *V. destructor* has been present since at least 1978, most of the colonies rely on acaricides for mite control (Invernizzi et al. 2011; Anido et al. 2015). Usually, beekeepers apply synthetic acaricides in autumn (March to June) and organic acaricides in early spring (September), but in recent years, treatments in both periods with oxalic acid and glycerine strips are commonly used (Maggi et al. 2016). However, there are regions on the country's eastern side where honey bees coexist with *V. destructor* without acaricide treatments. Research on these honey bee populations has been ongoing since 2011 (Invernizzi et al. 2016; Mendoza et al. 2020).

In this study, high‐throughput sequencing was used to characterise and compare the viral profile of *V. destructor‐*resistant and ‐susceptible *A. mellifera* colonies. Notably, both populations harbour communities with similar viral richness, diversity, and composition, as previously reported by Thaduri et al. (2018).

When considering the viruses known to be linked with honey bee diseases, ABPV showed a higher abundance in susceptible colonies in autumn. This finding might be related to a higher *V. destructor* infestation and a vulnerability to virus amplification in *V. destructor‐susceptible* colonies. ABPV is associated with honey bee colony losses, especially when colonies are also infected by *V. destructor* (reviewed by Yañez et al. 2020). Although there is no direct evidence of ABPV replication within varroa mites, high levels of ABPV have been found in individual varroa‐parasitized honey bees and entire honey bee colonies, suggesting that the mite could act as a vector for ABPV (Traynor et al. 2020; Yañez et al. 2020). Another explanation for the higher ABPV levels in susceptible colonies could be due to the *V. destructor* ‐sensitive hygienic behaviour of the resistant honey bees, which could identify ABPV‐infected pupae and remove them from the colony, decreasing the infecting virions levels (Mondet et al. 2016). This finding underscores the potential of resistant colonies to limit the spread or impact of viruses like the ABPV.

Our results also showed that the season was the main factor affecting virome diversity and composition rather than *V. destructor‐*resistant and ‐susceptible colony status. In autumn, viral diversity was higher than in spring. Previous studies also reported that the season affects viral infection levels (Thaduri et al. 2018; Beaurepaire et al. 2020; Chen et al. 2021). *Varroa destructor* ‐susceptible colonies harbour more reads of bacteria and insects‐non bee viruses in autumn, although no significant differences in overall viral richness and diversity between resistant and susceptible colonies were found. The seasonal variation in the viromes could be attributed to changes in colony dynamics, environmental conditions, and available forage, which can affect virus transmission and replication rates (Beaurepaire et al. 2020).

Metagenomic characterisation of Uruguayan honey bee viromes allowed us to obtain complete or partial genomes of the most studied honey bee viruses. In particular, we report partial and complete genomes of ABPV and SBV for the first time in South America. Moreover, we added seven DWV complete genomes and 12 partial or complete genomes of the BQCV to the NCBI database. These genomes, together with those already available from the region (Barriga et al. 2012; Hasegawa et al. 2023; Gonzalez et al. 2024), may serve as input for future phylogeographic studies to reconstruct the origins and spread of these viruses in the region.

The genetic analysis of ABPV strains from Uruguay revealed a high percentage of identity with strains from Brazil and Zimbabwe, forming a distinct phylogenetic clade separate from European and Asian strains. This may be associated with the African genetic origin of the samples, which could influence virus‐host dynamics. However, this hypothesis could not be tested due to the low number of ABPV genomes from Africanized honey bees in the NCBI database.

Regarding BQCV and DWV, most genomes cluster with Argentinian sequences, suggesting potential local adaptations or introductions of these viruses, as observed with ABPV. However, more sequences from South America and other continents are needed to perform robust phylogenetic studies. Besides that, this study confirms that circulating DWV strains correspond to DWV variant A, following previous studies (Hasegawa et al. 2023; Mendoza et al. 2020). This is the dominant variant in neighbouring South American countries such as Chile, Brazil, and Argentina (de Souza et al. 2019; Brasesco et al. 2021; Riveros et al. 2020; Gonzalez et al. 2024).

Additionally, LSV was detected for the first time in our country in several samples from resistant and susceptible honey bee colonies in autumn and spring. LSV has a positive‐sense, single‐stranded RNA genome and is taxonomically assigned to the genus *Sinaivirus*. Although the pathogenicity and specific symptoms of LSV infection in colonies have not been accurately elucidated yet, it is assumed to be an opportunistic pathogen to immunosuppressed honey bees infested with mites or infected with other viruses such as DWV (Doublet et al. 2024). The results indicate that, in our country, at least two of the six variants are circulating, regardless of whether the colonies are susceptible or resistant.

Finally, although this work focuses on RNA viruses, reads mapping to the AmFV were detected in two autumn samples, one from resistant colonies and the other from susceptible colonies. This virus is a large double‐stranded DNA linear virus with approximately 500 kilobases (kb) in length (Gauthier et al. 2015), geographically widespread (Quintana et al. 2019; Boncristiani et al. 2020; Lester et al. 2022; Papp et al. 2024). This is the first report of AmFV in Uruguay and the second in South America, following the finding of Quintana et al. (2019) in Argentina.

Complete genomes could not be obtained for several viruses, including ALPV, BeeMLV, IAPV, KBV, and VDV‐2. This limitation may be due to the overlap of short sequences among viral families, which can lead to misidentifications. In particular, we highlight that although some reads were initially identified as KBV, IAPV, or VDV‐2, further analysis led to their reclassification as ABPV or DWV, respectively.

In this initial study, we focused on a general characterisation of viromes by comparing seasonal differences and variations between *V. destructor* ‐resistant and susceptible colonies. Then, we focused on honey bee viruses that have been widely studied. However, the metagenomic analysis offers additional information that can be used to describe novel viruses, as reported by Da Assis Silva et al. (2023), Kadlečková et al. (2024), Kwon et al. (2024), Kwon et al. (2023), Mordecai et al. (2016) and Ryabov et al. (2023). Future studies will focus on identifying viruses not detected through reference‐based mapping. We will also explore the diversity of bacteriophages and their relationship with the gut microbiota, as described by Bonilla Rosso et al. (2020), Kadlečková et al. (2022) and Busby et al. (2022).

The findings of this study allow us to provide valuable insights into the honey bee virome and contribute to establishing a baseline for the understanding of the tripartite network involving *V. destructor* , honey bees, and viruses in resistant and susceptible colonies.

## Author Contributions

**Daniela Arredondo:** conceptualization, methodology, software, investigation, formal analysis, supervision, funding acquisition, visualization, project administration, resources, writing – original draft, writing – review and editing, data curation. **Sofia Grecco:** methodology, formal analysis, data curation, investigation, writing – review and editing. **Yanina Panzera:** methodology, software, supervision, formal analysis, resources, data curation, investigation, writing – review and editing. **Pablo Zunino:** conceptualization, writing – review and editing. **Karina Antúnez:** conceptualization, writing – original draft, writing – review and editing, resources.

## Ethics Statement

All authors have approved the content and authorship of the submitted manuscript, and all prevailing local, national, and international regulations and conventions and normal scientific ethical practices have been respected.

## Conflicts of Interest

The authors declare no conflicts of interest.

## Supporting information


**Data S1.** Supporting Information.

## Data Availability

The viral data obtained by sequencing that support the findings of this study are openly available in the GenBank under Bioproject PRJNA1199515. The complete and partial genomes were also submitted under accession numbers PQ810637‐PQ810643 and PV008135‐PV008140.
