# Supplementary material for: Honey Bee Viromes From Varroa destructor ‐Resistant and Susceptible Colonies
Source: Environ Microbiol Rep. 2025 May 10;17(3):e70097. doi: 10.1111/1758-2229.70097 (PMC12064942; doi:10.1111/1758-2229.70097)
Supplement: Supplementary file 1 — Data S1. Supporting Information. [file EMI4-17-e70097-s001.docx]

**Honey bee viromes from *Varroa destructor*-resistant and susceptible colonies**

Daniela Arredondo_ab_, Sofia Grecco_cd_, Yanina Panzera_cd_, Pablo Zunino_ab_, Antúnez, Karina_ab_

a Laboratorio de Microbiología y Salud de las Abejas, Departamento de Microbiología, Instituto de Investigaciones Biológicas Clemente Estable, Ministerio de Educación y Cultura, Av. Italia 3318, CP. 11600. Montevideo, Uruguay.

b Centro de Investigación en Ciencias Ambientales (CICA), Instituto de Investigaciones Biológicas Clemente Estable, Ministerio de Educación y Cultura, Av. Italia 3318, CP. 11600. Montevideo, Uruguay.

c Sección Genética Evolutiva, Departamento de Biología Animal, Facultad de Ciencias, Universidad de la República, Iguá 4225, CP. 11400. Montevideo, Uruguay.

d Plataforma de Genómica, Facultad de Ciencias, Universidad de la República, Iguá 4225, CP. 11400. Montevideo, Uruguay..

*Corresponding author: Daniela Arredondo danielarpapiol@gmail.com. Laboratorio de Microbiología y Salud de las Abejas, Departamento de Microbiología, Instituto de Investigaciones Biológicas Clemente Estable, Ministerio de Educación y Cultura, Av. Italia 3318, CP. 11600. Montevideo, Uruguay.

**Supplementary material**

**Table S1.** Number of reads obtained and filtered for each library. Nurse honey bees were sampled in September (spring) and March (autumn) from six *V. destructor*-resistant (R) and six susceptible (S) colonies located in the Treinta y Tres department (-33°15'20.147"S 54°25'37.081" W).

| Library | Total reads | Reads passed  QC filter | Reads unmapped Honey bee host |
| --- | --- | --- | --- |
| SS1 | 8372374 | 8288372 | 6329618 |
| SS2 | 7336854 | 7250206 | 5213036 |
| SS3 | 7187222 | 7067140 | 5920356 |
| RS1 | 5750936 | 5697992 | 2116376 |
| RS2 | 8716794 | 8647628 | 563604 |
| RS3 | 7859492 | 7783212 | 3392038 |
| SA1 | 6001706 | 5955432 | 3845068 |
| SA2 | 7041492 | 6993748 | 2722854 |
| SA3 | 8266810 | 8192192 | 3727632 |
| RA1 | 6166886 | 6141134 | 4603646 |
| RA2 | 7127126 | 7078284 | 3092324 |
| RA3 | 7557880 | 7465556 | 3051314 |
| **Average** | **7282131** | **7213408** | **3714822** |


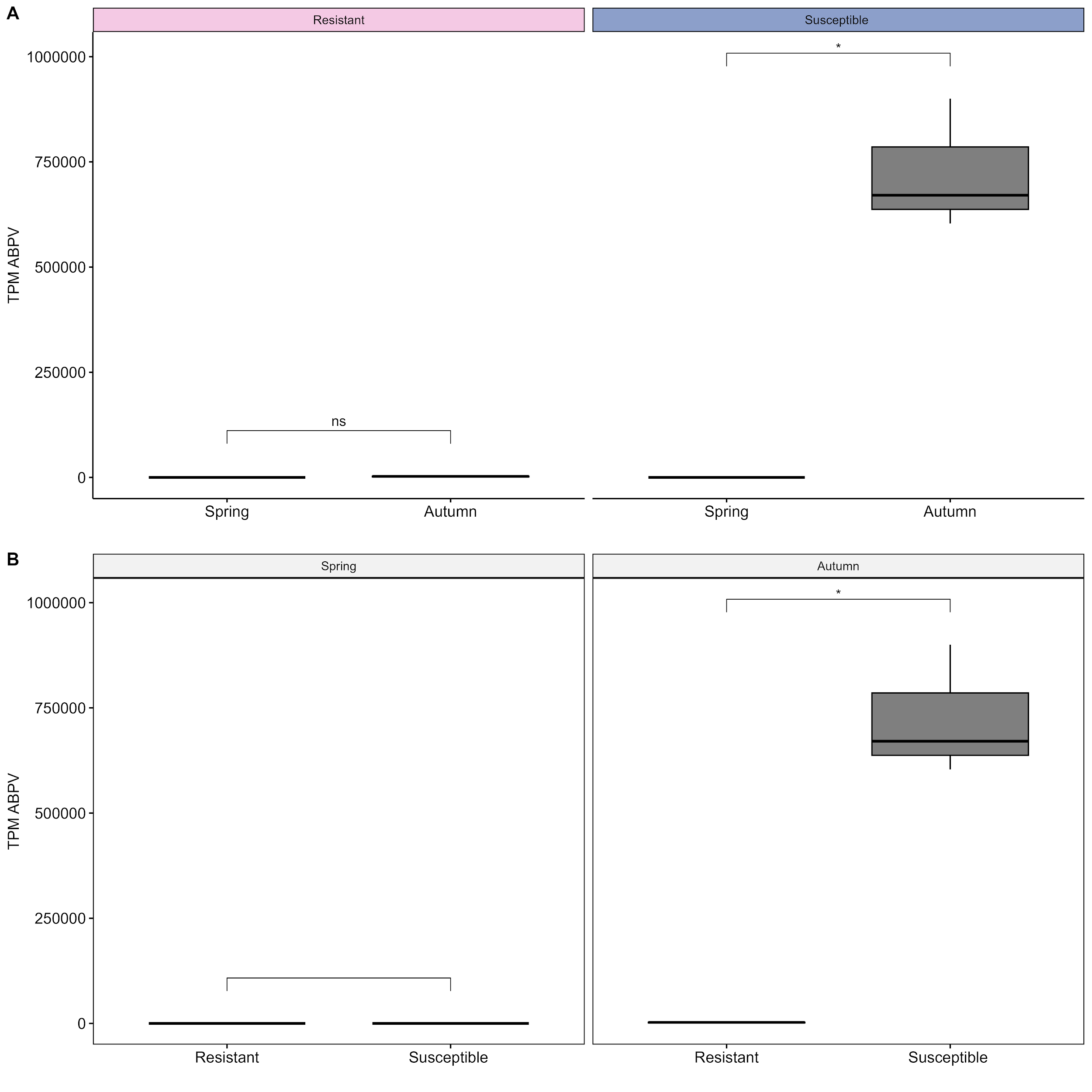


**Figure S1.** *ABPV* TPM counts. (A) Comparison within seasons (spring and autumn) (B) Comparison within the type of colony (tolerant and susceptible). The asterisks indicate statistically significant differences, as determined by Welch’s t-test (* = p < 0.05, ** = p < 0.01, *** = p < 0.001).
